# Running on Empty Signals: What Human MALT1 Deficiency Reveals About Immune Control

**DOI:** 10.1155/jimr/6626069

**Published:** 2026-09-15

**Authors:** Hulya Kose, Koray Yalcin, Elif Guler Kazanci

**Affiliations:** ^1^ Department of Pediatric Immunology and Allergy, University of Health Sciences, Bursa, Türkiye, akdeniz.edu.tr; ^2^ Department of Pediatric Hematology-Oncology and Stem Cell Transplantation Unit, Acibadem University, Istanbul, Türkiye, acibadem.edu.tr; ^3^ Department of Pediatric Hematology-Oncology, Health Science University, Bursa, Türkiye, sbu.edu.tr

**Keywords:** B cells, CBM complex, immune dysregulation, immunodeficiency, MALT1, regulatory T cells

## Abstract

Human genetic disorders affecting intracellular signaling pathways provide an unparalleled opportunity to understand how immune responses are regulated in vivo. Among these conditions, MALT1 deficiency has emerged as a particularly informative model because it reveals how subtle quantitative changes in antigen receptor signaling can translate into profound clinical consequences. As a central component of the CARD11–BCL10–MALT1 (CBM) signalosome, MALT1 integrates receptor‐derived signals and determines whether downstream nuclear factor kappa B (NF‐kB) activation reaches thresholds required for effective immune responses. Rather than representing a single, uniform loss‐of‐function condition, MALT1 deficiency is genetically and functionally heterogeneous: different pathogenic variants affect MALT1 protein expression, paracaspase/protease activity, or its scaffolding role within the CBM complex to varying degrees, and these differences determine whether the predominant phenotype reflects impaired immune activation, immune dysregulation, or a combination of both. This functional diversity helps explain why patients with MALT1 deficiency may present with recurrent infections, inflammatory features, regulatory T‐cell defects, and progressive impairment of B‐cell immunity, either alone or in combination. In this review, we discuss how insights from human disease, experimental models, and therapeutic studies converge to position MALT1 as a critical regulator of immune homeostasis and explore broader implications for translational immunology. Due to the rarity of MALT1 deficiency, we carefully compiled and contextualized the few reported cases of hematopoietic stem cell transplantation (HSCT), highlighting emerging translational patterns.

## 1. Introduction

The study of rare immune disorders has repeatedly reshaped our understanding of human immunology by revealing mechanisms that cannot be easily dissected through experimental systems alone. Genetic defects affecting intracellular signaling are particularly illuminating because they expose vulnerabilities within pathways that govern immune activation. In this context, the CARD11–BCL10–MALT1 (CBM) signalosome serves as a central regulatory hub, translating antigen recognition into transcriptional programs that determine immune cell behavior [1–4].

MALT1 occupies a unique position within this complex. Unlike many signaling molecules that function primarily as adaptors or enzymes, MALT1 performs both roles simultaneously. This dual functionality allows it to coordinate the assembly of signaling complexes while also modulating signal intensity through proteolytic processing of regulatory proteins [2, 5]. Such an arrangement suggests that MALT1 does not merely transmit signals but actively shapes the quality and duration of immune responses.

The emergence of human MALT1 deficiency has provided compelling evidence supporting this concept. Rather than simply impairing immune activation, disruption of MALT1 reveals how immune responses depend on the precise tuning of signaling thresholds. This insight has broad implications, extending beyond rare diseases to fundamental questions about immune tolerance, inflammation, and the therapeutic modulation of signaling pathways.

**Figure 1:**
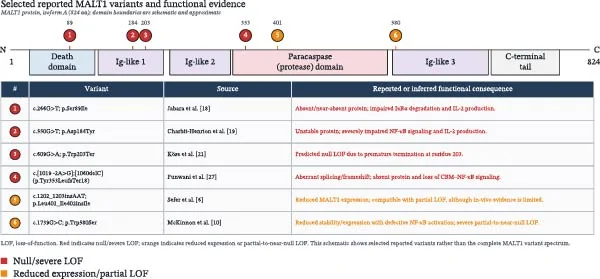
Representative reported MALT1 pathogenic variants mapped onto the main protein domains (schematic; domain boundaries approximate). Variants are classified by predominant functional consequence: complete loss‐of‐function (red) and partial loss‐of‐function/hypomorphic (orange).

Previous case series and reviews have substantially expanded our knowledge of the clinical manifestations and disease course of human MALT1 deficiency. However, how individual variant characteristics, remaining MALT1 activity, and downstream immune consequences interact to shape the final clinical phenotype remains an important unresolved translational question [6–8]. This mini‐review does not aim to repeat the published clinical case summaries; instead, it brings together evidence from patients and experimental models to clarify why different patterns of MALT1 dysfunction may result in related but nonidentical immune phenotypes. We specifically focus on how complete functional loss, hypomorphic disease, selective defects in protease activity, and impairment of MALT1’s scaffolding role may differentially affect conventional T‐cell responses, regulatory T‐cell function, B‐cell maintenance, and inflammatory regulation. Figure 1 summarizes representative reported MALT1 patient variants, mapped onto the main functional domains of the protein and classified by their predominant functional consequence (complete loss‐of‐function, partial loss‐of‐function/hypomorphic) to illustrate this genotype‐functional diversity before the individual mechanisms are discussed in detail below.

## 2. MALT1 as a Regulator of Signaling Amplitude

Activation of lymphocytes requires more than simple receptor engagement; it depends on coordinated signaling events that collectively determine whether the activation threshold is surpassed. The CBM complex orchestrates this process by linking receptor‐proximal signals to the activation of nuclear factor kappa B (NF‐kB) transcription factors [3, 4]. Within this network, MALT1 serves as a stabilizing scaffold that facilitates the assembly of signaling intermediates.

Equally important is the proteolytic activity of MALT1, which selectively cleaves negative regulators such as A20, thereby prolonging signaling and preventing premature termination of immune responses [2, 5]. This mechanism ensures that transient receptor engagement can, when appropriate, generate sustained transcriptional programs. From a systems perspective, MALT1 can therefore be viewed as a molecular amplifier that reinforces productive immune activation.

Beyond canonical signaling, MALT1 influences additional regulatory layers, including modulation of RNA stability and noncanonical NF‐kB pathways [9–13]. These functions collectively highlight the breadth of its regulatory influence and underscore why disruption of MALT1 has widespread immunological consequences.

## 3. Genotype–Functional Phenotype Relationships in MALT1 Deficiency

Human MALT1 deficiency shows considerable genetic and functional diversity. Variants that introduce premature stop codons, frameshifts, or protein truncation can markedly diminish MALT1 expression or eliminate the protein altogether, resulting in severe disruption of CBM complex–dependent NF‐kB signaling. By contrast, missense and hypomorphic variants may leave a degree of MALT1 expression or partial signaling capacity intact while still modifying the strength, persistence, or character of downstream immune activation [6, 8, 14]. This functional gradient is clinically important as the extent of preserved MALT1 activity may affect susceptibility to infections, the severity of immune dysregulation, and the pace of clinical progression.

For this reason, the impact of each MALT1 variant should be interpreted through several functional dimensions. A given variant may compromise protein stability or abundance, interfere with MALT1’s scaffolding function within the CBM complex, or selectively disrupt its paracaspase activity. Impairment of the protease function may, in turn, alter the cleavage of substrates that regulate NF‐kB signaling, RNA stability, and inflammatory pathways [4, 5, 12, 13, 15–17]. These effects may overlap in the same patient rather than acting as isolated mechanisms. Thus, the phenotype of MALT1 deficiency is best understood as the combined result of biallelic pathogenic variants, residual protein activity, and specific functional domains affected by the mutation.

## 4. Clinical Insights From Human MALT1 Deficiency

Patients with MALT1 deficiency illustrate how impaired signaling amplitude manifests clinically. Affected individuals typically present with recurrent bacterial, viral, and fungal infections early in life, reflecting compromised adaptive immune responses [6, 8, 10, 11, 18]. Despite relatively preserved lymphocyte counts in some patients, functional defects are evident, emphasizing that effective immunity depends not only on cell numbers but also on signaling competence.

Clinical observations further reveal that MALT1 deficiency is not limited to immunodeficiency. Some patients develop inflammatory manifestations or features of immune dysregulation, including dermatitis, enteropathy, autoimmune‐like manifestations, and failure to thrive, suggesting that inadequate or imbalanced signaling may disrupt mechanisms that maintain tolerance [6, 8, 11, 19]. This dual phenotype highlights the delicate balance between activation and regulation within the immune system.

The broad clinical spectrum of MALT1 deficiency can also be understood through its distinct effects on the effector and regulatory immune pathways. In conventional T cells, defective MALT1‐mediated signaling weakens antigen receptor‐driven activation, cellular expansion, cytokine generation, and overall adaptive immune function. At the same time, MALT1 is required for the development and suppressive capacity of regulatory T cells. When this regulatory arm is impaired, immune tolerance may be compromised, contributing to dermatitis, enteropathy, autoimmune‐like features, and systemic inflammatory manifestations [15, 17, 20]. These opposing consequences help explain why MALT1 deficiency may present with an apparently paradoxical combination of immune insufficiency and immune activation. The relative predominance of these features may vary depending on the extent of residual MALT1 function and whether the underlying variant primarily disrupts protein expression, scaffolding activity, protease activity, or multiple mechanisms.

Humoral immune defects are also central to the human phenotype. Patients with MALT1 deficiency may develop progressive B‐cell depletion, impaired B‐cell differentiation, reduced memory B‐cell populations, hypogammaglobulinemia, and inadequate antibody responses [6, 7]. These observations support a broader role for MALT1 beyond T‐cell immunity, extending to sustained B‐cell maintenance and effective antibody‐mediated immune protection.

Recent reports, including our own case describing a novel truncating mutation, have broadened the clinical spectrum and reinforced the concept that disease severity correlates with the degree of signaling impairment [6, 21]. Such observations support a model in which the residual signaling capacity modulates clinical presentation.

## 5. Protease Activity Versus Scaffolding Function

MALT1 contributes to immune signaling through two closely related but functionally distinguishable roles. On the one hand, it provides a structural platform within the CBM complex that supports signalosome assembly; on the other hand, it functions as a paracaspase/protease that processes selected substrates after antigen receptor engagement. When MALT1 is completely disrupted, both activities may be lost, resulting in impaired lymphocyte activation and combined immunodeficiency. In contrast, variants or experimental conditions that predominantly affect protease activity may produce a different immunological pattern. Studies in experimental models indicate that loss of MALT1 protease function can weaken immune responses while simultaneously favoring immune dysregulation and inflammatory pathology [15, 16]. Thus, MALT1 should be considered a fine regulator of immune signaling rather than a simple binary switch.

**Figure 2:**
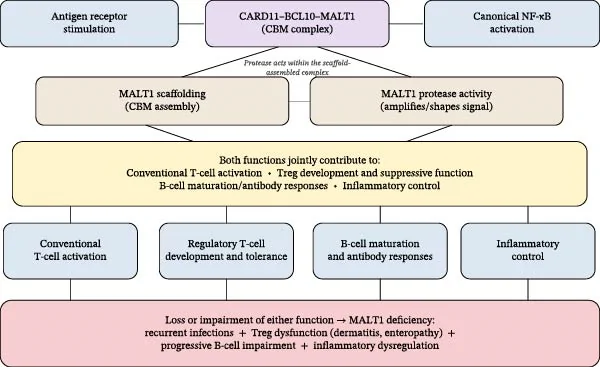
Functional consequences of MALT1 deficiency. MALT1 is a central component of the CARD11–BCL10–MALT1 complex; its scaffolding function assembles the signalosome that drives canonical NF‐kB activation, while its protease activity acts within this scaffold to amplify and shape the resulting signal. Both functions jointly, rather than separately, contribute to conventional T‐cell activation, regulatory T‐cell development and tolerance, B‐cell maturation and antibody responses, and inflammatory control; loss or impairment of either function can therefore contribute to the combined immunodeficiency and immune dysregulation seen in MALT1‐deficient patients.

This functional separation is particularly relevant for understanding human MALT1 deficiency. Variants that eliminate both scaffold‐dependent and protease‐dependent activities are expected to cause more profound immune failure, whereas variants that retain partial scaffolding function but disturb protease‐mediated regulation may allow some immune activation while predisposing to inflammation and immune imbalance. For this reason, future reports of MALT1‐deficient patients should ideally combine genetic findings with functional analyses, including assessment of MALT1 protein expression, NF‐kB pathway activation, protease activity, and cleavage of downstream substrates. Such data would strengthen genotype–phenotype interpretation and may support individualized therapeutic decisions, including the optimal timing of hematopoietic stem cell transplantation (HSCT). Figure 2 illustrates the proposed mechanisms underlying MALT1 deficiency, emphasizing that scaffolding and protease functions act together, rather than through separate nonoverlapping branches, to shape T‐cell, regulatory T‐cell, B‐cell, and inflammatory outcomes.

## 6. Lessons From Experimental Systems

Experimental studies have complemented clinical observations by elucidating the mechanistic basis of the MALT1 function. Mouse models lacking Malt1 exhibit impaired immune responses, confirming its essential role in NF‐kB activation [3, 22]. Interestingly, models with selective impairment of protease activity demonstrate inflammatory phenotypes, indicating that both insufficient and dysregulated signaling can lead to pathology. These findings reinforce the idea that immune signaling operates within a narrow physiological range. MALT1 helps maintain this balance by ensuring signals are neither too weak nor overly prolonged [16, 17, 22, 23]. From this perspective, MALT1 functions as a gatekeeper of immune homeostasis.

## 7. HSCT

HSCT has emerged as the definitive treatment for patients with severe MALT1 deficiency. Successful transplantation restores immune competence by replacing defective immune cells with donor‐derived cells that can function normally [7, 24–27]. Reported cases demonstrate improved infection control and immune reconstitution following transplantation. The outcomes summarized in Table 1 highlight the importance of early diagnosis and intervention [6, 7, 10, 18, 19, 21, 26, 27].

**Table 1:** MALT1 deficiency patients who underwent HSCT.

Study (year)	MALT1 variant type	Age at HSCT	Conditioning regimen	Donor type	Outcome
Rozmus et al., 2016 [26]	Homozygous c.1739G>C (p.Trp580Ser)	16 years	Not specified/reported as reduced intensity in some summaries	HLA‐matched sibling	Alive and functional immunological normalization
Punwani et al., 2015 [27]	Homozygous c.1019‐2A>G; de novo c.1059delC	18 months	Reduced intensity	9/10 matched unrelated donor	Mixed chimerism
Jabara et al., 2013 [18]	Homozygous c.266G>T	13 and 7 years	Myeloablative	Matched sibling donor	Immune reconstitution and alive
McKinnon et al., 2014 [10]	Homozygous truncating c.1739G>C (p.W580S)	1 year	Myeloablative	Matched related donor	Survived and infections improved
Charbit‐Henrion et al., 2017 [19]	Homozygous missense c.550G>T (p.D184Y)	2 and 4 years	Reduced intensity	Matched unrelated donor	Alive and partial immune recovery
Sefer et al., 2022 [6]	c.1202_1203insAAT (p.L401_402insI)	4 years	Reduced intensity	Matched unrelated donor	Alive
Kose, 2025 [21]	Homozygous nonsense c.609G>A (p.Trp203 ^∗^)	15 months	Reduced intensity	Matched related donor	Early follow‐up

Abbreviation: HSCT, hematopoietic stem cell transplantation.

Clinical experience demonstrates that transplantation leads to immune reconstitution and improved clinical outcomes, emphasizing the importance of early diagnosis. At the same time, pharmacologic inhibition of MALT1 has attracted interest as a therapeutic strategy in malignancies characterized by constitutive NF‐kB activation [28, 29]. The contrasting phenotypes observed in patients underscore the need for careful consideration when targeting this pathway therapeutically as excessive inhibition may compromise immune defense and immune regulation.

## 8. Broader Implications for Immune Regulation

The insights derived from MALT1 deficiency extend beyond a single disorder. They illustrate how quantitative regulation of signaling pathways determines immune outcomes and highlight the importance of threshold control in preventing both immunodeficiency and autoimmunity. Similar principles likely apply across multiple signaling networks within the immune system.

Within the broader category of CBM opathies, comparative analysis of related disorders has revealed shared mechanistic themes, including disrupted signal propagation and altered immune homeostasis [6, 10, 19, 30]. Studying these conditions collectively may provide a more comprehensive understanding of signaling regulation.

The study of MALT1 deficiency also has broader translational implications. Pharmacologic MALT1 inhibition is being explored in lymphoid malignancies and inflammatory disorders; however, human MALT1 deficiency demonstrates that excessive or prolonged inhibition of this pathway may compromise both immune defense and immune regulation. This concern is not merely theoretical: toxicology studies of a selective MALT1 protease inhibitor in rats and dogs documented a treatment‐related fall in circulating regulatory T cells and an evolving, multiorgan autoimmune picture resembling IPEX over weeks of dosing, echoing the tolerance defect associated with genetic protease loss and underscoring that the protease itself, not just the scaffold, is required to keep peripheral tolerance intact [31]. Therefore, lessons from rare human immunodeficiency, together with preclinical toxicology data, should inform the safety and biological interpretation of MALT1‐targeted therapies.

## 9. Future Perspectives

Although important advances have been made, several issues with direct clinical relevance remain to be clarified. Additional patients with well‐defined genetic variants and detailed functional evaluation are needed to understand how individual MALT1 mutations influence protein abundance, protease activity, scaffolding capacity, and downstream NF‐kB signaling. Harmonized functional testing will be particularly important for separating complete loss‐of‐function disease from hypomorphic or functionally selective forms. In addition, extended clinical follow‐up is required to better characterize the evolution of immune dysregulation, progressive B‐cell impairment, infection burden, and post‐HSCT outcomes.

Future investigations should go beyond purely descriptive case reports and include deeper immune phenotyping. This should involve the evaluation of T‐cell activation, regulatory T‐cell number and function, B‐cell subsets, immunoglobulin production, and MALT1‐dependent substrate cleavage. Such integrated analyses may help explain why some patients mainly develop severe infectious complications, whereas others show more prominent inflammatory or IPEX‐like features. They may also support more individualized therapeutic decisions, including identifying patients who require early transplantation and those in whom alternative targeted approaches might be considered in milder or hypomorphic diseases.

## 10. Conclusion

Human MALT1 deficiency demonstrates that impairment of a single intracellular signaling component can result in a broad immune phenotype characterized by susceptibility to infection, immune dysregulation, inflammatory manifestations, and progressive humoral immune defects. The variability observed among patients is likely influenced by the nature of the underlying MALT1 variant, the extent of residual protein function, and the degree to which protease‐dependent and scaffolding‐dependent activities are affected. When considered together, human observations and experimental data show that MALT1 is a critical regulator of immune signaling thresholds that helps preserve the balance between protective immunity and immune tolerance. A clearer definition of genotype–functional phenotype relationships will therefore be important for more accurate diagnosis, risk assessment, and individualized treatment planning in patients with MALT1 deficiency.

## Author Contributions

Hulya Kose conceived the commentary, conducted the literature review, integrated genetic and mechanistic insights, and wrote the manuscript. Koray Yalcin and Elif Guler Kazanci contributed clinical expertise related to hematopoietic stem cell transplantation and critically reviewed the manuscript for clinical accuracy.

## Funding

The authors received no specific funding for this work.

## Disclosure

All authors have approved the final version of the manuscript.

## Ethics Statement

Written informed consent was obtained from the parents.

## Conflicts of Interest

The authors declare no conflicts of interest.

## Data Availability

The data are available upon reasonable request from the corresponding author.
